# Maternal effects shape the alternative splicing of parental alleles in reciprocal cross hybrids of *Megalobrama amblycephala* × *Culter alburnus*

**DOI:** 10.1186/s12864-020-06866-7

**Published:** 2020-07-02

**Authors:** Li Ren, Xiaojing Yan, Xin Gao, Jialin Cui, Pengcheng Yan, Chang Wu, Wuhui Li, Shaojun Liu

**Affiliations:** 1 State Key Laboratory of Developmental Biology of Freshwater Fish, Hunan Normal University, Changsha, 410081 Hunan P.R. China; 2 College of Life Sciences, Hunan Normal University, Changsha, 410081 Hunan P.R. China; 3 Tang Tang Biomedical Technology (BeiJing) Co., Ltd., Beijing, P.R. China

**Keywords:** Maternal effects, Alternative splicing, Reciprocal cross hybridization, Differential expression, Homoeologous expression

## Abstract

**Background:**

Maternal effects contribute to adaptive significance for shaping various phenotypes of many traits. Potential implications of maternal effects are the cause of expression diversity, but these effects on mRNA expression and alternative splicing (AS) have not been fully elucidated in hybrid animals.

**Results:**

Two reciprocal cross hybrids following hybridization of *Megalobrama amblycephala* (blunt snout bream, BSB) and *Culter alburnus* (topmouth culter, TC) were used as a model to investigate maternal effects. By comparing the expression of BSB- and TC- homoeologous genes between the two reciprocal cross hybrids, we identified 49–348 differentially expressed BSB-homoeologous genes and 54–354 differentially expressed TC-homoeologous genes. 2402, 2959, and 3418 AS events between the two reciprocal cross hybrids were detected in Illumina data of muscle, liver, and gonad, respectively. Moreover, 21,577 (TC-homoeologs) and 30,007 (BSB-homoeologs) AS events were found in the 20,131 homoeologous gene pairs of TBF_3_ based on PacBio data, while 30,561 (TC-homoeologs) and 30,305 (BSB-homoeologs) AS events were found in BTF_3_. These results further improve AS prediction at the homoeolog level. The various AS patterns in *bmpr2a* belonging to the bone morphogenetic protein family were selected as AS models to investigate the expression diversity and its potential effects to body shape traits.

**Conclusions:**

The distribution of differentially expressed genes and AS in BSB- and TC-subgenomes exhibited various changes between the two reciprocal cross hybrids, suggesting that maternal effects were the cause of expression diversity. These findings provide a novel insight into mRNA expression changes and AS under maternal effects in lower vertebrates.

## Background

Maternal effects are the causal influence of the maternal genotype or phenotype on the phenotype of the offspring [1]. The maternal influence is generally in the form of maternal messenger RNAs that are partly made by maternal mitochondrial genes and shape the traits of hybrids including growth and starvation resistance, similar to that of maternal parents [2, 3]. The definition of maternal effects is often extended to incorporate a diversity of other related phenomena (e.g. kin effects, genomic imprinting, uniparental extra-chromosomal inheritance) [1]. Some studies reported that the maternal effects associated with methyltransferase led to maternal genomic imprinting [4, 5], which referred to the phenomenon where individuals expressed only one copy of the maternal or paternal allele. More generally, it refers to parent-of-origin-dependent gene expression or effects [6, 7]. Although biologists have known about the importance of these effects for decades, their influences on expression diversity in offspring have not been fully elucidated.

Alternative splicing (AS), including skipped exons (SE), retained introns (RI), alternative 5′ splice sites (A5SS), alternative 3′ splice sites (A3SS), and alternative position (AP), generates multiple transcripts from the same gene by combining different exons. It expands transcriptome plasticity and proteomic diversity, thereby regulating gene expression at the post-transcriptional level [8]. Pre-mRNA splicing is largely co-transcriptional, and the alternative splice site choice is influenced by the RNA polymerase II elongation rate, chromatin remodelers, and histone deacetylase inhibitors [9, 10]. Recent studies using Illumina and PacBio sequencing indicated that about 25, 60, and 90% of multi-exon genes in *Caenorhabditis elegans*, *Drosophila melanogaster*, and human, respectively, undergo AS [11–13]. Changes in AS represent one of the major driving forces underlying the evolution of phenotypic differences across different species [14, 15]. However, few studies have focused on hybrids because of their complex regulation patterns [16].

Homoeologs are the orthologous gene pairs from two or more inbred hybrid parents in allodiploids and allopolyploids. The unequal expression of two or more homoeologs (also described as homoeolog expression bias), and the total expression level of a homoeolog pair similar to one of the two diploid parents (also described as expression level dominance), are contributed to the formation of various phenotypes, including heterosis [17–19]. Studies on homoeologs provide us a insight into the potential regulation mechanisms of various phenotypes. However, unlike in plants, intergeneric allodiploids are rarely found in vertebrates because of reproductive isolation and chromosomal pairing disorder during gamete formation, or hybrid individuals with failed to have offspring. However, two intergeneric reciprocal cross hybrids were previously obtained by the hybridization of *Megalobrama amblycephala* (BSB) and *Culter alburnus* (TC) [20, 21], which exhibited slight differences in body shape. In the present study, we detected differences in global expression (in both of two alleles) and homoeologous expression (in each of two alleles) between two reciprocal cross hybrids. We also predicted AS differences between the two homoeologs based on Illumina and PacBio data. Our results provide a comprehensive study of regulatory divergence under maternal effects.

## Results

### Origin of reciprocal cross hybrids

We first characterized the divergence of AS between two reciprocal cross hybrids (BTF_3_ and TBF_3_), which were obtained from the self-crossing of respective reciprocal cross hybrids of *M. amblycephala* (2n = 48) × *C. alburnus* (2n = 48) [20, 21]. The genotype of chimeric offspring was determined as the allodiploid (2n = 48) with a 1:1 subgenome ratio with chromosome number and 45S rDNA characteristics [20], in which the two types of 45S rRNA were detected and belonged to species-specific sequences of *M. amblycephala* and *C. alburnus*, respectively [21] (Additional file 1**: Table S1**). The expression of mitochondrial genes in the two reciprocal cross hybrids was considered to be identical to that of the respective inbred female parents based on the mapped reads of transcriptome (Additional file 2**: Table S2**).

### Characteristic differences of the two subgenomes

For the two inbred parental genomes (1.09 Gb in BSB and 1.02 Gb in TC), the distributions of exon numbers and CDS lengths were obtained from 20,131 orthologous gene pairs (Additional file 3**: Fig. S1**). The average exon number in each gene was 8.83 in BSB and 9.64 in TC, while the average CDS length was 1525 bp in BSB and 1654 bp in TC. Focusing on same characteristic in the two parental genomes, the same exon number was found in 11,414 genes, and the same CDS length was detected in 6832 genes. Analysis of these results showed significant differences in exon number (*p* < 0.001) and CDS length (*p* < 0.001). Furthermore, strong associations with exon number and CDS length were detected in BSB (*r* = 0.7435) and TC (*r* = 0.7768) (all *p* < 0.0001 for Pearson correlation coefficients).

### Obtaining of long length transcripts and gene ontology analysis

PacBio sequencing was used to detect AS events in reciprocal cross hybrids. A total of 21.22 Gb and 15.49 Gb data were obtained from TBF_3_ and BTF_3_, respectively, and an average of 12 and 13 CCSs and 3080 bp and 2936 bp average insert read lengths were detected in TBF_3_ and BTF_3_, respectively (Table 1). To detect the integrity of sequencing data, 663,834 TBF_3_ and 479,667 BTF_3_ 3′ and 5′- untranslated regions were analyzed to determine whether the transcripts were full-length. Then, 586,075 (88.29%) and 431,999 (90.06%) full-length reads were detected in TBF_3_ and BTF_3_ (Table 1). After deleting redundant sequences, a total of 316,533 and 268,986 consensus reads were found in TBF_3_ and BTF_3_, respectively.

**Table 1:** Summary of full-length transcriptome data

	TBF_3_	BTF_3_
The sequencing data (Gb)	21.22	15.49
Insert reads (Gb)	2.02	1.30
Average length of insert read (bp)	3080.18	2936.23
Average CCSs of insert reads	12	13
Number of consensus reads	663,834	479,667
Number of five prime reads	622,119 (93.72%)	459,029 (95.70%)
Number of three prime reads	628,065 (94.61%)	456,107 (95.09%)
Number of full-length reads	586,075 (88.29%)	431,999 (90.06%)

After mapping to the combined genome of the two inbred parents, the 314,298 consensus reads and 76,518 isoforms were obtained from the mapped results of TBF_3_, while 267,949 consensus reads with 82,083 isoforms were found in BTF_3_. An average 99.29 and 99.61% of mapping ratios were detected in TBF_3_ and BTF_3_, respectively. Then, the sequences of 11,026 genes in TBF_3_ and 11,448 genes in BTF_3_ were annotated with KEGG and GO databases (Additional file 4**: Fig. S2**). The 6071 genes shared between TBF_3_ and BTF_3_ were then focused on to help detect differences between the two.

### AS between two homoeologs

**Fig. 1:**
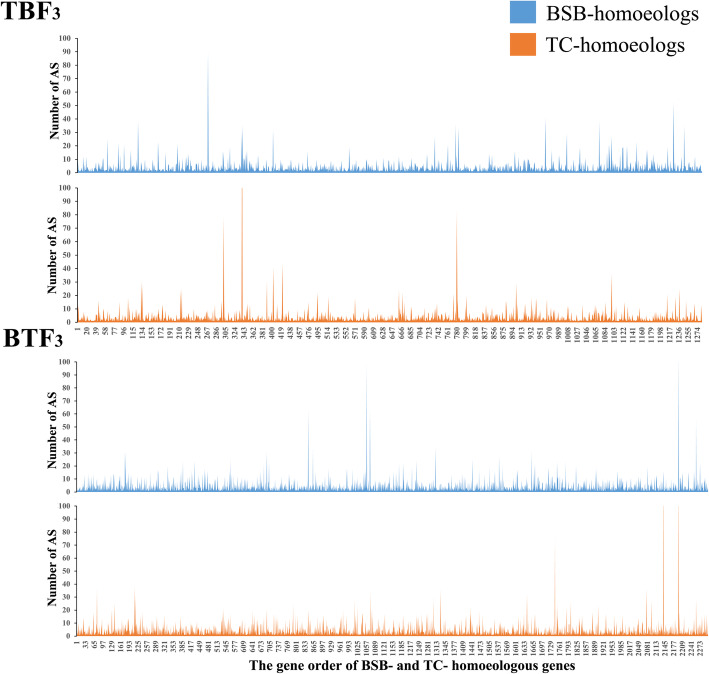
Distribution of AS events observed in orthologous gene pairs of the two reciprocal cross hybrids. The difference on AS number was shown between BSB- and TC- homoeologs in each orthologous gene pairs

To better characterize the differences between TBF_3_ and BTF_3_, we focused on AS events in BSB- and TC- homoeologs of the two reciprocal cross hybrids. A custom Python script was used to identify 30,007 AS events, and 7029 genes were mapped to the BSB-subgenome detected in 20,131 homoeologous genes of TBF_3_, while 21,577 AS events and 5286 genes were mapped to the TC-subgenome (Table 2). We also detected 30,305 AS events and mapped 7271 genes to the BSB-subgenome of BTF_3_, while 30,562 AS events related to 6481 genes were mapped to the TC-subgenome (Table 2). Although the sequencing was performed in a mixture of three tissues, these data suggested a slight BSB-homoeolog expression bias in TBF_3_ but not in BTF_3_. Most of the AS events that occurred in hybrids were RI in TC-homoeologs of TBF_3_ (18.96%) and BTF_3_ (26.49%), and BSB-homoeologs of BTF_3_ (26.70%). However, most AS events in BSB-homoeologs of TBF_3_ were SE (20.28%) (Table 2). Although some errors in gene annotation may have led to an increased prediction of RI and SE, these results suggest that there are clear differences not only between the two reciprocal cross hybrids, but also between BSB- and TC-homoeologous genes. Then, we compared the number of AS events between in each orthologous gene pairs of two subgenomes. Among these, 1290 genes of TBF_3_ and 2302 genes of BTF_3_ were shown to possess AS events in both homoeologs. In TBF_3_, 4862 AS events supported by 6416 isoforms were mapped to the BSB-subgenome, while 4650 AS events supported by 6674 isoforms were mapped to the TC-subgenome (Fig. 1). In addition, we detected the 8054 AS events shared in orthologous gene pairs of BSB-subgenome and TC-subgenome of TBF_3_, while the 11,024 AS events were shared in ones of BTF_3_.

**Table 2:** Summary of AS in BSB- and TC- homoeologs from full-length transcriptome data

AS types	TC-homoeologs in TBF_3_		BSB-homoeologs in TBF_3_		TC-homoeologs in BTF_3_		BSB-homoeologs in BTF_3_	
	NO. of events (%)	NO. of gene	NO. of events (%)	NO. of gene	NO. of events (%)	NO. of gene	NO. of events (%)	NO. of gene
Alternative 3′ splice Site	1430 (6.63%)	988	2026 (6.75%)	1439	1714 (5.61%)	1177	1877 (6.19%)	1374
Alternative 5′ splice Site	1603 (7.43%)	1140	2384 (7.94%)	1625	1912 (6.26%)	1271	2122 (7.00%)	1533
Alternative site	2520 (11.68%)	1409	3300 (11.00%)	1640	4001 (13.09%)	1912	3538 (11.67%)	1899
Exon skipping	3869 (17.93%)	2137	6086 (20.28%)	3379	3685 (12.06%)	2050	5101 (16.83%)	3005
Retained Introns	4091 (18.96%)	823	5140 (17.13%)	1842	8096 (26.49%)	3107	8092 (26.70%)	3181
Other	8064 (37.37%)	2001	11,071 (36.89%)	2745	11,153 (36.49%)	2491	9575 (31.60%)	2861
Total	21,577	5286	30,007	7029	30,561	6481	30,305	7271

### Expression changes led by maternal effects

**Fig. 2:**
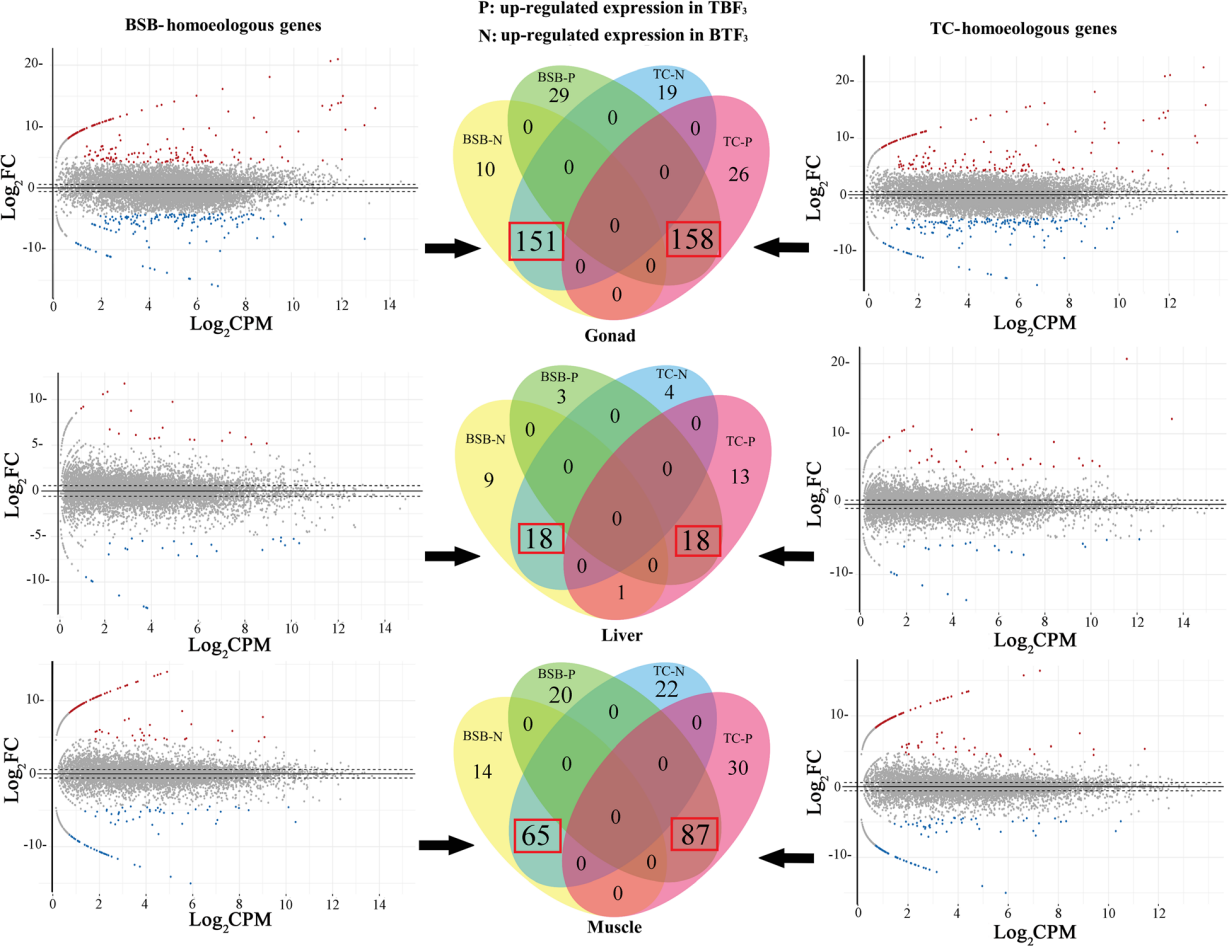
Detection of DEGs in two homoeologs of BSB- and TC- subgenomes. Comparison with BTF_3_ and TBF_3,_ differential expression analysis was performed in two homoeologs of BSB- and TC- subgenomes, respectively. “red dot” represents the up-regulated expressed gene in TBF_3_, while “blue dot” represents the up-regulated expressed gene in BTF_3_. Shared DEGs are distributed in Venn diagram. The most number of DEGs (red box) were shared in BSB- and TC- homoeologous genes, reflecting the same regulation mechanisms occurred in both of them. The values of log_2_ fold change (FC) and log_2_ counts per million (CPM) were used to assess significant DEGs

Comparison between BTF_3_ and TBF_3_, we identified 49 differentially expressed genes (DEGs) in BSB-homoeologous genes of liver, 186 DEGs in muscle, and 348 DEGs in gonad; this compared with 54 DEGs in TC-homoeologous genes of liver, 204 in muscle, and 354 in gonad (Fig. 2**,** Additional file 5**: Table S3**). The largest number of DEGs was found in gonad (3.58% in BSB-homoeologs and 3.64% in TC-homoeologs) and the fewest were found in liver (0.50% in BSB-homoeologs and 0.56% in TC-homoeologs) (Fig. 2**,** Additional file 5**: Table S3**).

We next focused on DEGs that were shared between BSB- and TC-homoeologs. The same up/down-regulated expression trends of the two homoeologs were exhibited among the three tissues (Fig. 2**,** Additional file 5**: Table S3**), indicating that similar differential expression trends occurred in both homoeologs. GO analysis showed that 90, 12, and 51 DEGs (the largest number in GO categories) were involved in the cellular process (GO: 0009987) (level 2) in gonad, liver, and muscle tissues, respectively (Additional file 6**: Fig. S3 and S4**). Some DEGs were also enriched in other functions, including metabolic process (GO: 0008152), response to stimulus (GO: 0050896), and biological regulation (GO: 0065007), while others were enriched in growth (GO: 0040007), immune system process (GO: 0002376), and reproduction (GO: 0000003) (Additional files 6 and 7**: Fig. S3 and S4**).

### Determination of AS in DEGs

To further investigate the maternal effects on expression divergence, AS analysis was performed in homoeologous genes between the reciprocal cross hybrids. ASprofile detected 104 MXE and 3314 SE in gonad, 96 MXE and 2863 SE in liver, and 74 MXE and 2328 SE in muscle (Additional file 8**: Table S4**). Interestingly, 3103 (90.78%) AS events were found in TC-homoeologous genes of gonad, while 315 (9.22%) AS events were found in BSB-homoeologous genes. Moreover, 2706 (91.45%) AS events were distributed in TC-homoeologous genes of liver, and the remaining 253 (8.55%) AS events occurred in BSB-homoeologous genes. In muscle, 2205 (91.80%) AS events occurred in TC-homoeologous genes compared with 197 (8.20%) AS events in BSB-homoeologous genes (Additional file 8**: Table S4**). However, no RI, A5SS, or A3SS were identified in Illumina data. A total of 41, 31, and 22 genes were detected as high AS events (number of AS types ≥5 in each gene) from muscle, liver, and gonad, respectively. Among these, the five genes (*ptprm*, *cast*, *exoc*, *myo1b*, and *abi1a*) with a high number of AS events were shared among the three tissues (Additional file 9**: Fig. S5**).

Combined analyses of AS and DEG, we identified AS events in 35 DEGs in gonad, 18 DEGs in muscle, and six DEGs in liver. Under different maternal effects, changes to homoeologous gene expression and AS events were found in reciprocal cross hybrids. However, the details of some AS events were inaccurate using Illumina data because of the short length of the reads. Therefore, long length reads of BTF_3_ PacBio data were used to improve the analysis of AS events in DEGs, including 38 AS events in gonad, 16 in muscle, and two in liver, while TBF_3_ data improved AS events in 33, 14, and six DEGs in gonad, muscle, and liver, respectively.

### AS distribution in bone morphology

**Fig. 3:**
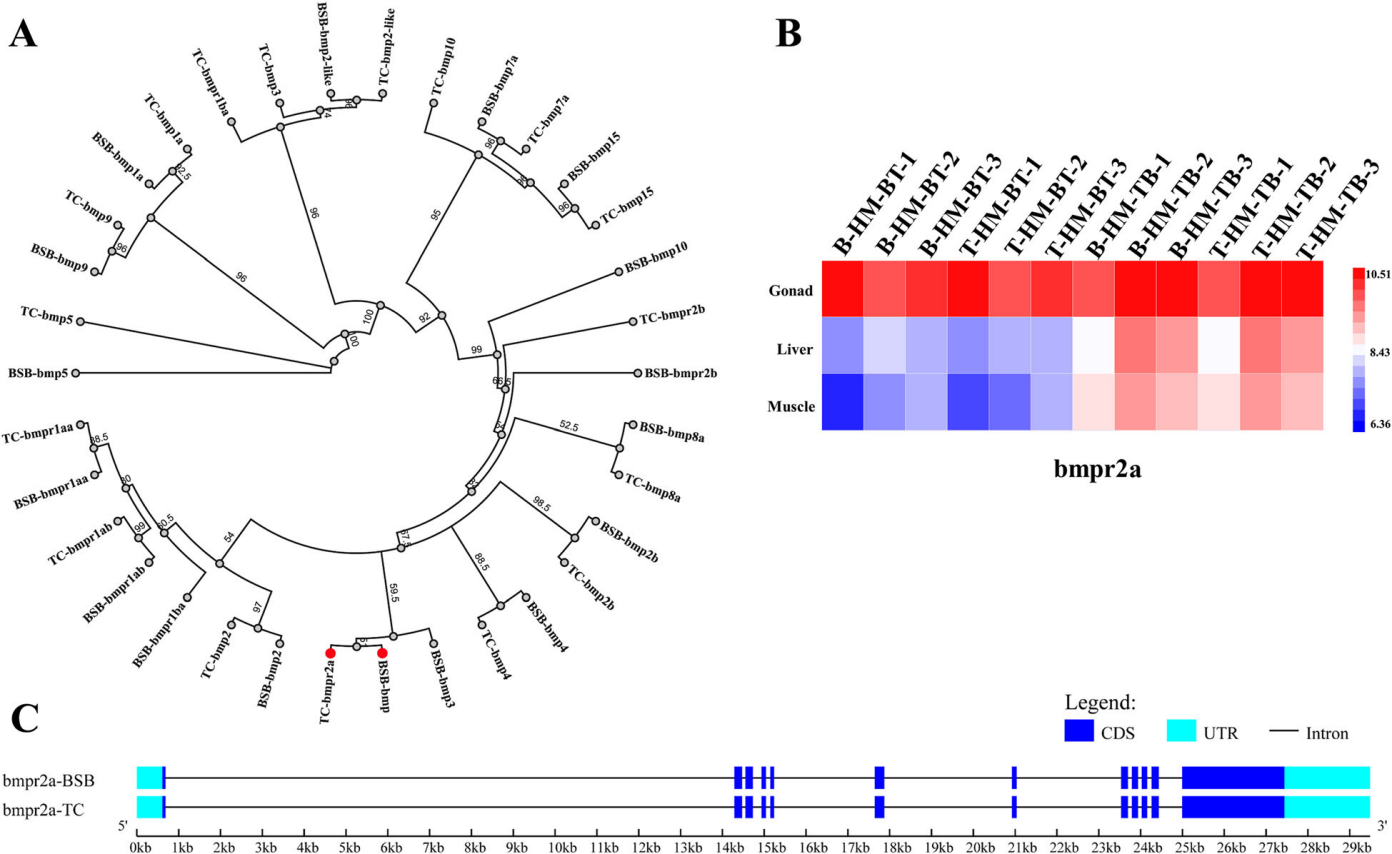
Phylogenetic tree of the bone morphogenetic protein (BMP) family and homoeolog expression of *bmpr2a*. **a** Phylogenetic neighbor-joining tree of the BMP family between *M. amblycephala* (BSB) and *C. alburnus* (TC). The genetic distance model was used with the Tamura–Nei method [22] and bootstraps were shown around corresponding branches. **b** Heatmap showing the homoeolog expression of *bmpr2a*, which was not significant different between TBF_3_ and BTF_3_ in all three tissues. **c** The gene structure of *bmpr2a*

**Fig. 4:**
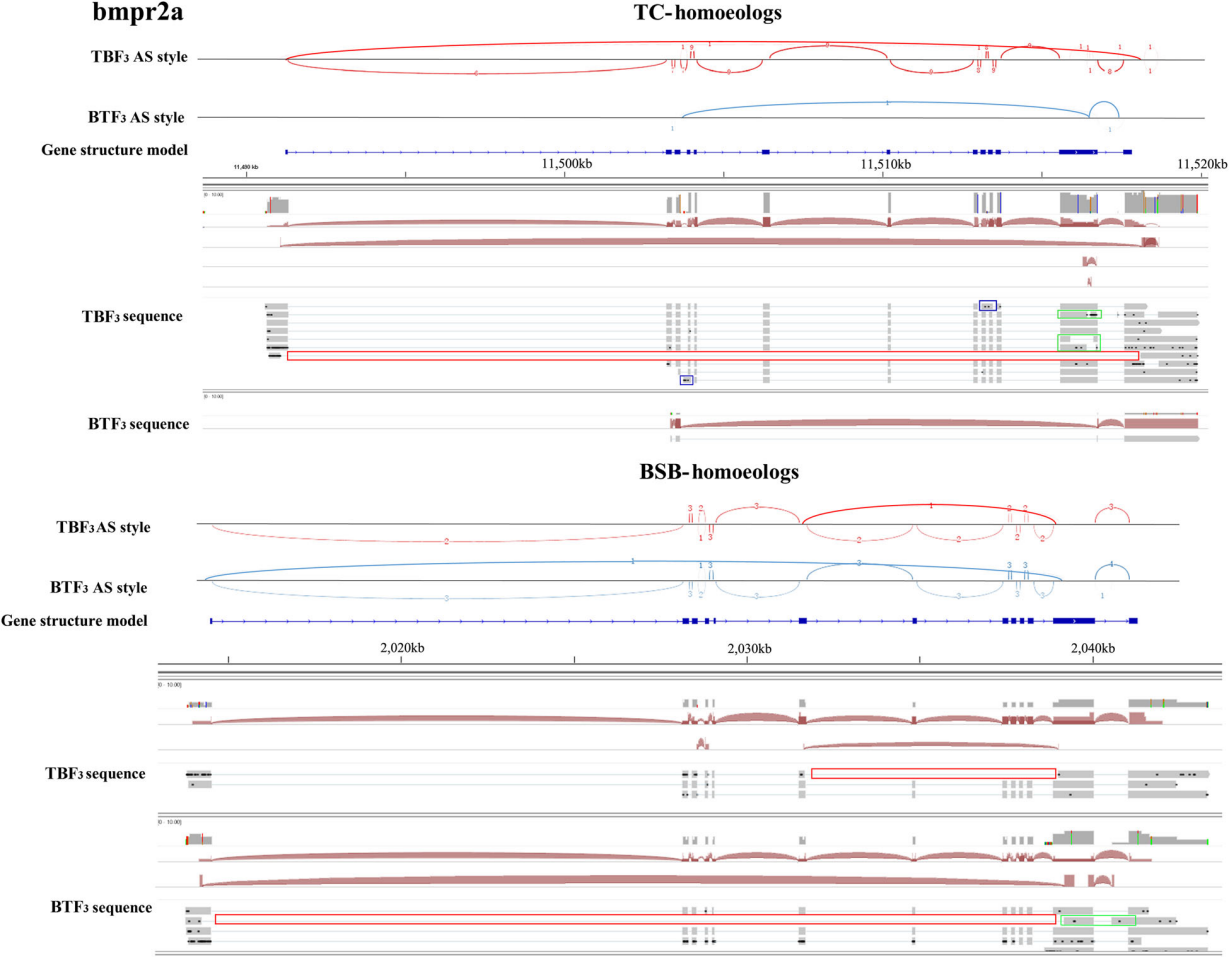
Various AS events detected in BSB- and TC- homoeologs of *bmpr2a*. Red box represents skipped exons (SE), blue box represents retained introns (RI), and the green box represents alternative 3′ splice site (A3SS) events

In view of the many shared traits between the reciprocal cross hybrids, the observed slight differences in their appearance made us consider their control of bone morphology regulated gene expression. Focusing on the BMP family, 17 orthologous genes were obtained from BSB and TC genomes, which exhibited gene expansion events (Fig. 3a). However, BSB- and TC- homoeologous gene expression in the three tissues studied was only detected in *bmpr2a* simultaneously. Slight differences in homoeologous gene expression were observed between TBF_3_ and BTF_3_ (Fig. 3b), although these were not significant. Therefore, *bmpr2a* was selected as a model to investigate AS events in BSB- and TC- homoeologs. We identified 12 exons, which was identical to the zebrafish (Fig. 3c) [23]. SE differences of two, one, and zero were detected between TBF_3_ and BTF_3_ in gonad, muscle, and liver, respectively, using Illumina data, but fewer AS events were detected in PacBio data. However, longer length transcripts provided more accurate AS predictions in respective BSB- and TC- homoeologs. In TC- homoeologs of *bmpr2a*, two RI events were observed between exons 9 and 10 and between exons 3 and 4 in TBF_3_, while three A3SS events and one SE were detected (Fig. 4). Unfortunately, we only detected one isoform because of the potential sequencing bias. In BSB-homoeologs of *bmpr2a*, one SE event was identified that led to the loss of sequences in exons 7–11 of TBF_3_. Interestingly, we also identified an SE event with the loss of sequences in exons 2–11 in BTF_3_, similar to the SE event in the TC-homoeologous gene of TBF_3_ (Fig. 4).

For further determination of AS events in *bmpr2a*, the transcripts in the muscle of TB and BT were sequenced by Sanger method. The one RI event between exons 9 and 10 (AS_2) and the two SE events distributed in parts of exon 12 (AS_3 and 4) were detected in TC-homoeologs of BTF_3_ and TBF_3_. These phenomena were same to the above results of PacBio data (Fig. 4; Additional file 10**: Fig. S6**). Furthermore, one SE in exon 7 (AS_1) was observed in BSB-homoeologs of BTF_3_ and TBF_3_ (Additional file 10**: Fig. S6**).

## Discussion

Hybrid, especially intergeneric hybrid, is a useful model to investigate homoeologs because of the more specific loci and SNPs that differ between the two subgenomes. Although plants have a large number of allodiploids and allopolyploids, the rarity in lower vertebrate species hinders our study of their expression. The establishment of two reciprocal cross hybrids of *Megalobrama amblycephala* and *Culter alburnus* (2n = 48), with the same chromosome numbers as their inbred parents, provides a useful model to study maternal effects, especially to regulation of mitochondrial DNA. It enabled us to obtain 20,131 species-specific orthologous gene pairs between *M. amblycephala* and *C. alburnus*.

Maternal effects may arise through mitochondrial DNA, cytoplasmic factors in the transmission of organelles, maternal environmental effects and so on [24, 25]. Our study only focused on the regulation of mitochondrial DNA. In the two reciprocal cross hybrids, mitochondria of the two species could lead to the different regulation pattern on energy metabolism by mitochondrial gene expression, and further change the growth characteristic, including body shape traits [26]. The slight differences in bone morphology between BTF_3_ and TBF_3_ provided us with an insight into the potential regulation of maternal effects. This represents an important field of study in evolutionary ecology, and there is an ongoing debate regarding their adaptive significance which acts to increase offspring fitness [27]. Here, we captured the expression diversity under the maternal effects of *M. amblycephala* and *C. alburnus*. In a comparison of the two reciprocal cross hybrids, TC-homoeologous genes exhibited slightly more differential expression than BSB-homoeologous genes (Fig. 2). This indicated that the maternal effect shaped the expression of both homoeologous genes, although there were few differences in DEGs between BSB- and TC- homoeologs. Furthermore, these results also showed that the maternal effects exhibited the different magnitudes in liver, muscle, and gonad. GO analysis of DEGs revealed that maternal effects could shape growth and immune functions by regulating corresponding gene expression.

AS is one of the most important components of genome functional complexity [28], and the resulting multiple transcripts lead to an abundance of gene expression profiles [8]. We identified 2402, 2959, and 3418 AS events between the two reciprocal cross hybrids in muscle, liver, and gonad, respectively, and PacBio sequencing resulted in a more accurate AS prediction, obtaining 76,518 isoforms in TBF_3_ and 82,083 in BTF_3_. The difference on AS number in each or orthologous gene pairs reflected the maternal effects contributed to AS changes (Fig. 1). These AS differences under maternal effects suggested various potential mechanisms. The analysis of human embryoid bodies revealed that the expression of histone deacetylase was regulated by maternal effects [29], while distinctive histone modification caused splice site switching by influencing the recruitment of splicing regulators via a chromatin-binding protein [10, 30]. Furthermore, DNA methylation regulated the AS of mRNA precursors through two different mechanisms, including the elongation of RNA polymerase II by CCCTC-binding factor and methyl-CpG binding protein 2 [31]. On the other hand, expression divergence of homoeologs led to various expression patterns, including homoeolog expression bias and expression level dominance), further contributing to the formation of various phenotypes, including heterosis [17–19]. However, identification of homoeolog expression patterns with the corresponding phenotypes are challenging because of the complex gene regulatory network and unknown interactions between RNAs and proteins [32]. Studies on AS events on different homoeologs could help us gradually understanding of complex interactions among regulatory networks, in which AS events exhibits a close relationship with *cis*-regulation divergence [33].

Focusing on AS and expression in the BMP family in our study, we investigated the relationship of gene diversity and phenotypic changes in body shape between two reciprocal cross hybrids (Figs. 3 and 4). BMPs are involved in a host of cellular functions including osteogenesis, cell growth, and cell differentiation [34]. Among the family members, *bmpr2a* encodes a transmembrane serine/threonine kinase that plays an important role in follicle development in preparation for ovulation [35]. The ratio of multiple alternative spliced variants of isoform-A (full-length) to isoform-B (missing exon 12) regulates heritable pulmonary arterial hypertension in humans [36], although it may not be the main effect in the morphological difference. Except full-length isoform in *bmpr2a*, we further detected seven other isoforms in the TC- homoeologous gene, but only one in the BSB- homoeologous gene because of the low sequencing coverage. Therefore, additional PacBio sequencing data of hybrids and their inbred parents are required for future studies. Nevertheless, our results provide evidence of how maternal effects shape expression diversity.

## Conclusions

In this work, we focused on the changes of mRNA expression and AS related to maternal effects in the two reciprocal cross hybrids. First, the 49–348 differentially expressed BSB-homoeologous genes and 54–354 differentially expressed TC-homoeologous genes were detected between the two reciprocal cross hybrids. Second, 2402, 2959, and 3418 AS events were detected in three tissues (muscle, liver, and gonad) between the two reciprocal cross hybrids, respectively. PacBio data further improved AS prediction of Illumina data. Third, we detected various AS patterns in *bmpr2a*, which exhibited their potential effects to body shape between the two reciprocal cross hybrids. These findings provide a novel insight into mRNA expression changes and AS under maternal effects in lower vertebrates.

## Methods

### Animal materials

Fertile reciprocal cross hybrids (2n = 48) were obtained from hybridization between *M. amblycephala* × *C. alburnus*, and F_2_ and F_3_ generation offspring were obtained from the self-crossing of F_2_ and F_3_ reciprocal cross offspring [20, 21]. The allodiploid lineage then continued into F_5_ by successive self-crossing. Among these hybrid offspring, only chimeric offspring with half of the two inbred parental genomes (1:1) identified with 45S rDNA were used in our study [21]. These fishes were cultured to 2 years of age under the same suitable environment, including water temperature, dissolved oxygen content and adequate forage at the Engineering Center of Polyploidy Fish Breeding of the National Education Ministry, Hunan Normal University, Hunan, China. After collecting, the six reciprocal cross hybrids of F_3_ (three individuals in BT and three individuals in TB) were kept in the same aquarium (23 °C) for 2 days. Then, these fishes were deeply anaesthetized with 100 mg/L MS-222 (Tricaine Methanesulfonate) (Sigma-Aldrich, St Louis, MO, USA) for 10 min (25 °C) in a separate tank. Then, the more powder of MS-222 was added and mixed with water in the tank for increasing concentration to 300 mg/L. After checking the death of the fishes, they was moved from water for dissection. All tissue samples were excised carefully and subsequently stored at − 80 °C. These experiments were conducted at the Engineering Center of Polyploidy Fish Breeding of the National Education Ministry, Hunan Normal University, Hunan, China, and conformed to the National Institutes of Health Guide for Care and Use of Laboratory Animals. The animal work was approved by the academic committee in Hunan Normal University (approval ID: 01/2018).

Illumina reads of BTF_3_, TBF_3_, and their inbred parents (*M. amblycephala* and *C. alburnus*) as well as liver, muscle, and gonad tissues were downloaded from the NCBI Sequence Read Archive database (accession numbers: SRS404403, SRS4069048, SRS47368, and SRS805925). Raw reads containing adapters or ploy-N and low-quality reads were removed using in-house perl scripts. High-quality reads were used in subsequent analysis.

### Characteristics of two inbred parental genomes

Assembled genome of the two inbred parents (BSB and TC) help understand their gene structures (accession number: PRJNA269572 in NCBI BioProject database). Although lacking the genomes of hybrid offspring, we used the combined genomes representing the in silico genomes of hybrids. Exon numbers and the number and length of coding sequences (CDS) of the two genomes were annotated with Kyoto Encyclopedia of Genes and Genomes (KEGG) and Gene Ontology (GO) databases. Orthologous gene pairs between BSB and TC were determined by the best reciprocal BLAST hits with each other with an e-value of 1e^− 5^. The Ks value between the orthologous pairs was calculated by the yn00 program in PAML package. Then, the 17 orthologous gene pairs of bone morphogenetic protein (BMP) family were obtained based on the previous annotation results.

### Mapping of RNA sequencing (RNA-seq) data

All Illumina reads of *M. amblycephala* and *C. alburnus* were aligned to *M. amblycephala* and *C. alburnus* genomes using Star (v 2.4.0) with default parameters [37]. mRNA-seq reads of TB and BTF_3_ were aligned to the combined nuclear and mitochondrial genomes of *M. amblycephala* (accession number: MF522177.1) and *C. alburnus* (accession number: KX244762.1). The numbers of mapping counts in each gene were calculated with in-house perl scripts. To avoid the negative effect of expression noise, expression analysis was only performed on filtered genes with mapping read counts ≥5 in all three biological replicates in each contrast. The total expression value was normalized to the ratio of the number of mapped reads at each gene to the total number of mapped reads for the entire genome. Orthologous genes between *M. amblycephala* and *C. alburnus* were obtained from all–against–all reciprocal BLASTP (v 2.2.26) comparisons with the parameters of “-e 10-5 –F –v 1 –m 8” based on protein sequences. Transcripts lacking annotated CDSs and those of length < 100 bp were discarded.

### Detection of homoeologous gene expression in reciprocal cross hybrids

To investigate the expression level of parent-of-origin genes (homoeologous gene expression), the LASTZ pairwise alignment tool (v 1.02.00) [38] with default parameters was used to obtain corresponding loci from the orthologous gene pairs between *M. amblycephala* and *C. alburnus*. InDel loci were discarded, and only aligned loci with the best scoring match were used for the next analysis. Single nucleotide polymorphisms (SNPs) were collected using the SNP Calling pipeline with GATK (v 3.8) based on the results of parent transcriptome mapping to respective genomes. According to the comparison of SNPs and other loci in orthologous gene pairs between *M. amblycephala* and *C. alburnus*, loci with complete differences including heterozygous and homozygous loci were considered to be species-specific SNPs as seen in Schaefke et al [39, 40]. To remove the negative effect of sequencing and mapping, the screening of species-specific SNPs was checked for consistency in three biological replicates, and loci possessing read counts ≥1 in all accessions and biological replicates in each contrast were retained.

To describe homoeologous gene expression in hybrids, hybrid mapping results (bam files) of transcriptomes were used in our next analysis. The mapping files of each hybrid were divided into two categories based on parent reference genomes. BSB−/TC- homoeolog reads in the hybrid had been calculated using in-house perl scripts, based on corresponding BSB−/TC- species-specific SNPs in their corresponding mapping files. The expression levels of parents in homoeolog analysis were also counted based on respective reference genomes related to species-specific SNPs. To remove the negative effect of hybrid mutation sites in biological replicates, abnormal values were discarded when estimating BSB- and TC- homoeolog expression levels if the mapping reads of the species-specific SNP did not comply with the threshold of mean ± 2SD in three biological replicates. Then, the sum counts of BSB- and TC- homoeologs in each gene were normalized based on the ratio of the number of mapped reads at each gene to the total number of mapped reads for the entire genome [41]. The sum of BSB−/TC- homoeolog reads in all species-specific SNPs of each gene was used to assess BSB−/TC- homoeolog expression. Differentially expressed genes (DEGs) were analyzed with the edgeR package of the R program (version 2.13) (R Foundation for Statistical Computing, Vienna, Austria). A false discovery rate threshold ≤0.05 was used in the analysis.

### AS analysis of Illumina data

After classifying the two homoeologs (BSB and TC), we further analyzed AS events in TBF_3_ and BTF_3_ using Illumina data. Mapped reads were assembled to transcripts using Cufflinks software (v2.2.1). Transcripts from three biological replicates were then merged together. AS events in two respective homoeologs were predicted using ASprofile [42], and five AS events (mutually exclusive exons [MXE], SE, RI, A5SS, and A3SS) were detected in the above analysis pipeline.

### RNA isolation, library construction, and PacBio sequencing

To sequence the transcriptomes of reciprocal cross hybrids and their inbred parents, total RNA was isolated and purified by TRIzol LS reagent (Invitrogen Corp.,). The RNA concentration was measured using a Nanodrop spectrophotometer. Total RNA samples were treated with DNase I (Invitrogen) to remove any contaminating genomic DNA. The purified RNA was quantified using a 2100 Bioanalyzer system (Agilent, Santa Clara, CA, USA).

To analyze AS in reciprocal cross F_3_ hybrid**s,** non-assembled long-length reads underwent PacBio sequencing. Total RNA of TBF_3_ and BTF_3_ from liver, muscle, and gonad was mixed in equal amounts. It was then reverse-transcribed using the SMARTer PCR cDNA Synthesis Kit and PCR-amplified using KAPA HiFi PCR kits (Kapa Biosystems). PCR products 0.5–6 kb and > 6 kb were selected using agarose gel electrophoresis. Libraries were then constructed from these cDNA products using the SMRTbell Template Prep Kit 1.0. After library preparation, the library template and enzyme mixture were used for sequencing in the PacBio Sequel™ system.

### AS analysis of PacBio data

After obtaining the sequencing data, adaptors were deleted using SMRTLINK (v 5.0.1) [43, 44]. Low-quality data (such as adaptor sequences, subreads < 50 bp, and reads with accuracy rates < 0.75) were deleted from the raw data. Sequence reads from the PacBio RS SMRT chip were processed using the PacBio SMRT-Portal analysis suite to generate circular consensus sequences (CCSs). To obtain greater accuracy, the reads (CCS cycles > 1 and accuracy > 0.8) were used to obtain full-length reads (> 300 bp, poly (A) tails, 5′ primers, and 3′ primers) based on the SMRT Iso-Seq analysis pipeline (http://www.pacb.com/products-and-services/analytical-software/smrt-analysis/). Full and reduced length transcripts were determined with both the m^7^G-cap structure and the poly(A) tail. Then, redundant sequences were clustered using the ICE algorithm, and consensus sequences were obtained by pbdagcon (https://github.com/PacificBiosciences/pbdagcon) with a Directed Acyclic Graph Consensus algorithm. Illumina reads of reciprocal cross hybrids were used to rectify sequencing errors using Lordec [45].

The above data were aligned to the combined reference genome sequences of *M. amblycephala* and *C. alburnus* using default parameters of GMAP software [46]. A custom Python script (alternative_splice.py, https://github.com/Nextomics/pipeline-for-isoseq) was used to identify AS events from alignments which were classified into MXE, SE, RI, A5SS, and A3SS. To obtain homoelogue AS in BT and TBF_3_, we focused on orthologous gene pairs between *M. amblycephala* and *C. alburnus*. Species-specific SNPs obtained from mRNA-seq data were used to rectify the mapping results of PacBio sequencing data. The threshold of parental homoelogue sequences was 85% of species-specific SNPs. Under this condition, the expression of BSB−/TC- homoeologs in BT and TBF_3_ was exhibited by the Integrative Genomics Viewer [47].

### AS determination in *bmpr2a* using sanger data

For checking the AS events in *bmpr2a*, total RNA extracted from muscle of BTF_3_ and TBF_3_ was treated with gDNA Eraser (TaKaRa). Then, first-strand cDNA was synthesized using AMV reverse transcriptase (Fermentas, Canada Inc.) with an oligo (dT)_12–18_ primer. The primers (5′- TTGAAGGCCGAATAACAATTCTT-3′, 3′-AGTCATTAGGATCTGAGAAGCGAG-5′) used to amplify the cDNA fragment of *bmpr2a* by PCR. The length of transcripts were exhibited by agarose gel electrophoresis. PCR-amplified products were separated on a 1.2% agarose gel using TBE buffer. The targeted fragments were purified using a gel extraction kit (Sangon,Shanghai, China) and ligated into the pMD18-T vector (TaKaRa, Dalian, China). The plasmids were transformed into E.coli DH5α and purified. The inserted targeted fragments in the pMD18-T vector were sequenced using Sanger sequencing. AS events were analyzed with BioEdit [48] and Clustal W [49].

## Supplementary information

**Additional file 1: Table S1.** The summary of 45S rDNA in the two reciprocal cross hybrids.

**Additional file 2: Table S2.** The determination of maternal expression of mitochondrial gene in the two reciprocal cross hybrids.

**Additional file 3: Figure S1.** Distribution of gene lengths and exon numbers.

**Additional file 4: Figure S2.** Shared genes between the two reciprocal cross hybrids detected by PacBio sequencing.

**Additional file 5: Table S3.** Differential expression between the two reciprocal cross hybrids detected in gonad, liver and muscle.

**Additional file 6: Figure S3.** Gene ontology (GO) categories (level 2) of DEGs in gonad, liver, and muscle.

**Additional file 7: Figure S4.** The distribution of DEGs in the biological process category (level 3) of gene ontology (GO).

**Additional file 8: Table S4.** The AS events and their gene distribution predicted from Illumina data.

**Additional file 9: Figure S5.** Distribution of genes with the high AS events (AS number ≥ 5) in gonad, liver, and muscle.

**Additional file 10: Figure S6.** AS events of *bmpr2a* detected by Sanger sequencing. (A) The model of AS events in TBF_3_ and BTF_3_. TC and BSB represent the complete coding sequences in parental TC and BSB, respectively. AS_1 is the SE event in BSB-homoeologs, while AS_2, 3 and 4 are the AS events in TC-homoeologs. (B) The sequence alignments of the complete coding sequences in TC and BSB and the four AS in TBF_3_ and BTF_3_.

## Data Availability

Raw data of PacBio sequencing have been submitted to the NCBI sequence read archive under accession numbers SRX5043671 and SRX5043670. Illumina reads of BTF_3_, TBF_3_, and their inbred parents (*M. amblycephala* and *C. alburnus*) as well as liver, muscle, and gonad tissues were downloaded from the NCBI Sequence Read Archive database (accession numbers: SRS404403, SRS4069048, SRS47368, and SRS805925). Assembled genomes of *M. amblycephala* and *C. alburnus* were downloaded from NCBI BioProject database (accession number: PRJNA269572). The mitochondrial genomes of *M. amblycephala* and *C. alburnus* were downloaded from accession numbers of MF522177.1 and KX244762.1 in NCBI Nucleotide Database, respectively.

## Abbreviations

CCSs: Circular consensus sequences
MXE: Mutually exclusive exons
DEGs: Differentially expressed genes
SNPs: Single nucleotide polymorphisms
RNA-seq: RNA sequencing
KEGG: Kyoto Encyclopedia of Genes and Genomes
GO: Gene Ontology
CDS: Coding sequences
BSB: *Megalobrama amblycephala*
TC: *Culter alburnus*
BT and TB: Two reciprocal cross hybrids of *M. amblycephala* and *C. alburnus*
AS: Alternative splicing
SE: Skipped exons
RI: Retained introns
A5SS: Alternative 5’ splice sites
A3SS: Alternative 3’ splice sites
AP: Alternative position
