# Supplementary material for: Maternal effects shape the alternative splicing of parental alleles in reciprocal cross hybrids of Megalobrama amblycephala × Culter alburnus
Source: BMC Genomics. 2020 Jul 2;21:457. doi: 10.1186/s12864-020-06866-7 (PMC7330940; doi:10.1186/s12864-020-06866-7)
Supplement: Supplementary file 1 — Additional file 1: Table S1. The summary of 45S rDNA in the two reciprocal cross hybrids. [file 12864_2020_6866_MOESM1_ESM.docx]

**Additional file 1: Table S1**. The summary of 45S rDNA in the two reciprocal cross hybrids.

|  | BSB specific 45S rDNA | TC specific 45S rDNA | Total 45S rDNA |
| --- | --- | --- | --- |
| BT_F_1__L1 | 5 | 5 | 10 |
| BT_F_1__L2 | 6 | 4 | 10 |
| BT_F_1__L3 | 4 | 6 | 10 |
| BT_F_2__L1 | 5 | 5 | 10 |
| BT_F_2__L2 | 5 | 5 | 10 |
| BT_F_2__L3 | 5 | 5 | 10 |
| BT_F_3__L1 | 5 | 5 | 10 |
| BT_F_3__L2 | 7 | 3 | 10 |
| BT_F_3__L3 | 4 | 6 | 10 |
| TB_F_1__L1 | 4 | 6 | 10 |
| TB_F_1__L2 | 6 | 4 | 10 |
| TB_F_1__L3 | 5 | 5 | 10 |
| TB_F_2__L1 | 5 | 5 | 10 |
| TB_F_2__L2 | 5 | 5 | 10 |
| TB_F_2__L3 | 4 | 6 | 10 |
| TB_F_3__L1 | 6 | 4 | 10 |
| TB_F_3__L2 | 3 | 7 | 10 |
| TB_F_3__L3 | 5 | 5 | 10 |
