# Supplementary material for: Maternal effects shape the alternative splicing of parental alleles in reciprocal cross hybrids of Megalobrama amblycephala × Culter alburnus
Source: BMC Genomics. 2020 Jul 2;21:457. doi: 10.1186/s12864-020-06866-7 (PMC7330940; doi:10.1186/s12864-020-06866-7)
Supplement: Supplementary file 2 — Additional file 2: Table S2. The determination of maternal expression of mitochondrial gene in the two reciprocal cross hybrids. [file 12864_2020_6866_MOESM2_ESM.docx]

**Additional file 2: Table S2**. The determination of expression of maternal mitochondrial gene in the two reciprocal cross hybrids.

| Sample | Mapped of TC mitochondrial genome (%) | Mapped of BSB mitochondrial genome (%) |
| --- | --- | --- |
| BSB-L1 | 3928 (0.15%) | 2,689,524 (99.85%) |
| BSB-L2 | 2472 (0.27%) | 920,772 (99.73%) |
| BSB-L3 | 2532 (0.11%) | 2,205,004 (99.89%) |
| BT_F_1__L1 | 17,602 (0.63%) | 2,789,256 (99.37%) |
| BT_F_1__L2 | 2258 (0.22%) | 1,014,342 (99.78%) |
| BT_F_1__L3 | 2188 (0.16%) | 1,346,972 (99.84%) |
| BT_F_2__L1 | 14,916 (0.65%) | 2,281,756 (99.35%) |
| BT_F_2__L2 | 1378 (0.22%) | 633,862 (99.78%) |
| BT_F_2__L3 | 2032 (0.30%) | 672,330 (99.70%) |
| BT_F_3__G1 | 1782 (0.37%) | 481,244 (99.63%) |
| BT_F_3__G2 | 1048 (1.03%) | 100,434 (98.97%) |
| BT_F_3__G3 | 1146 (1.14%) | 99,050 (98.86%) |
| BT_F_3__L1 | 1594 (0.37%) | 432,866 (99.63%) |
| BT_F_3__L2 | 1272 (0.28%) | 447,802 (99.72%) |
| BT_F_3__L3 | 1086 (0.23%) | 464,878 (99.77%) |
| BT_F_3__M1 | 824 (0.17%) | 484,438 (99.83%) |
| BT_F_3__M2 | 856 (0.14%) | 591,586 (99.86%) |
| BT_F_3__M3 | 614 (0.14%) | 428,786 (99.86%) |
| TB_F_1__L1 | 1,280,034 (99.75%) | 3234 (0.25%) |
| TB_F_1__L2 | 695,452 (99.87%) | 882 (0.13%) |
| TB_F_1__L3 | 2,329,286 (99.92%) | 1780 (0.08%) |
| TB_F_2__L1 | 1,139,286 (99.73%) | 3068 (0.27%) |
| TB_F_2__L2 | 842,768 (99.91%) | 750 (0.09%) |
| TB_F_2__L3 | 842,616 (99.91%) | 730 (0.09%) |
| TB_F_3__G1 | 293,924 (99.68%) | 948 (0.32%) |
| TB_F_3__G2 | 646,246 (99.73%) | 1778 (0.27%) |
| TB_F_3__G3 | 463,200 (99.72%) | 1284 (0.28%) |
| TB_F_3__L1 | 405,842 (99.75%) | 1026 (0.25%) |
| TB_F_3__L2 | 504,562 (99.40%) | 3036 (0.60%) |
| TB_F_3__L3 | 386,148 (99.56%) | 1692 (0.44%) |
| TB_F_3__M1 | 1,036,876 (99.82%) | 1862 (0.18%) |
| TB_F_3__M2 | 460,900 (99.61%) | 1818 (0.39%) |
| TB_F_3__M3 | 1,337,386 (99.80%) | 2620 (0.20%) |
| TC-L1 | 1,822,634 (98.48%) | 28,184 (1.52%) |
| TC-L2 | 530,600 (99.73%) | 1430 (0.27%) |
| TC-L3 | 421,454 (99.69%) | 1308 (0.31%) |
