# Supplementary figures and images for: Maternal effects shape the alternative splicing of parental alleles in reciprocal cross hybrids of Megalobrama amblycephala × Culter alburnus
Source: BMC Genomics. 2020 Jul 2;21:457. doi: 10.1186/s12864-020-06866-7 (PMC7330940; doi:10.1186/s12864-020-06866-7)

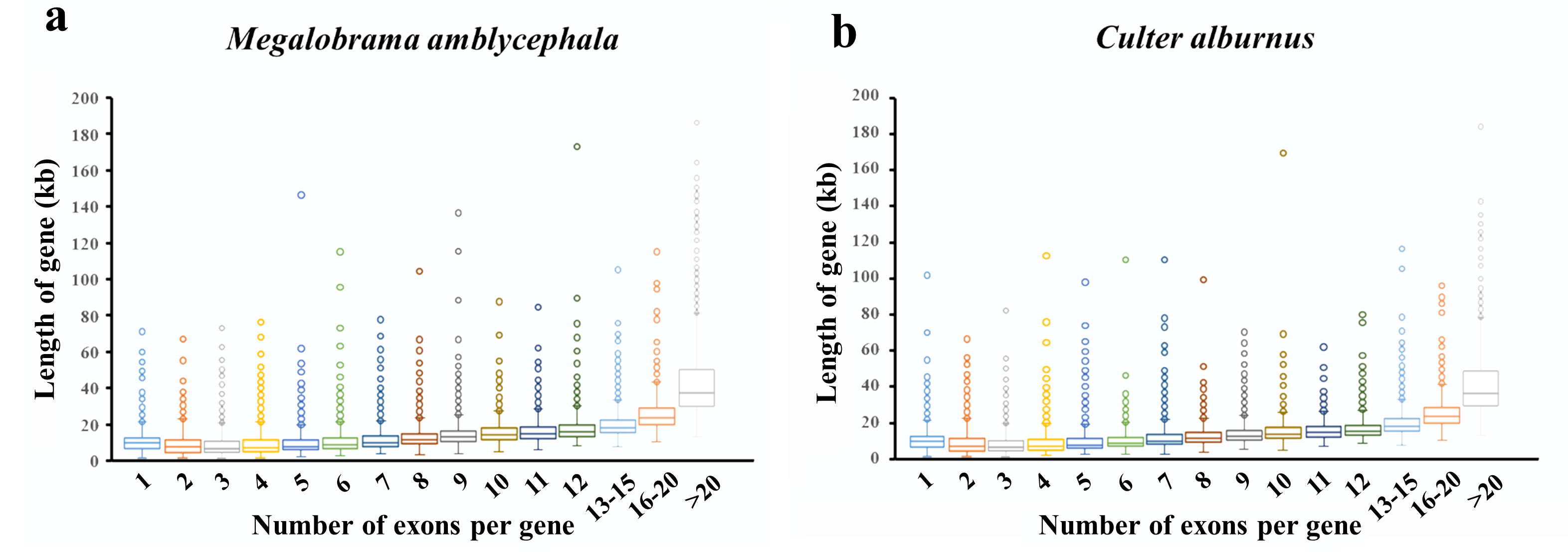

Supplement: Supplementary file 3 — Additional file 3: Figure S1. Distribution of gene lengths and exon numbers. [file 12864_2020_6866_MOESM3_ESM.tif]

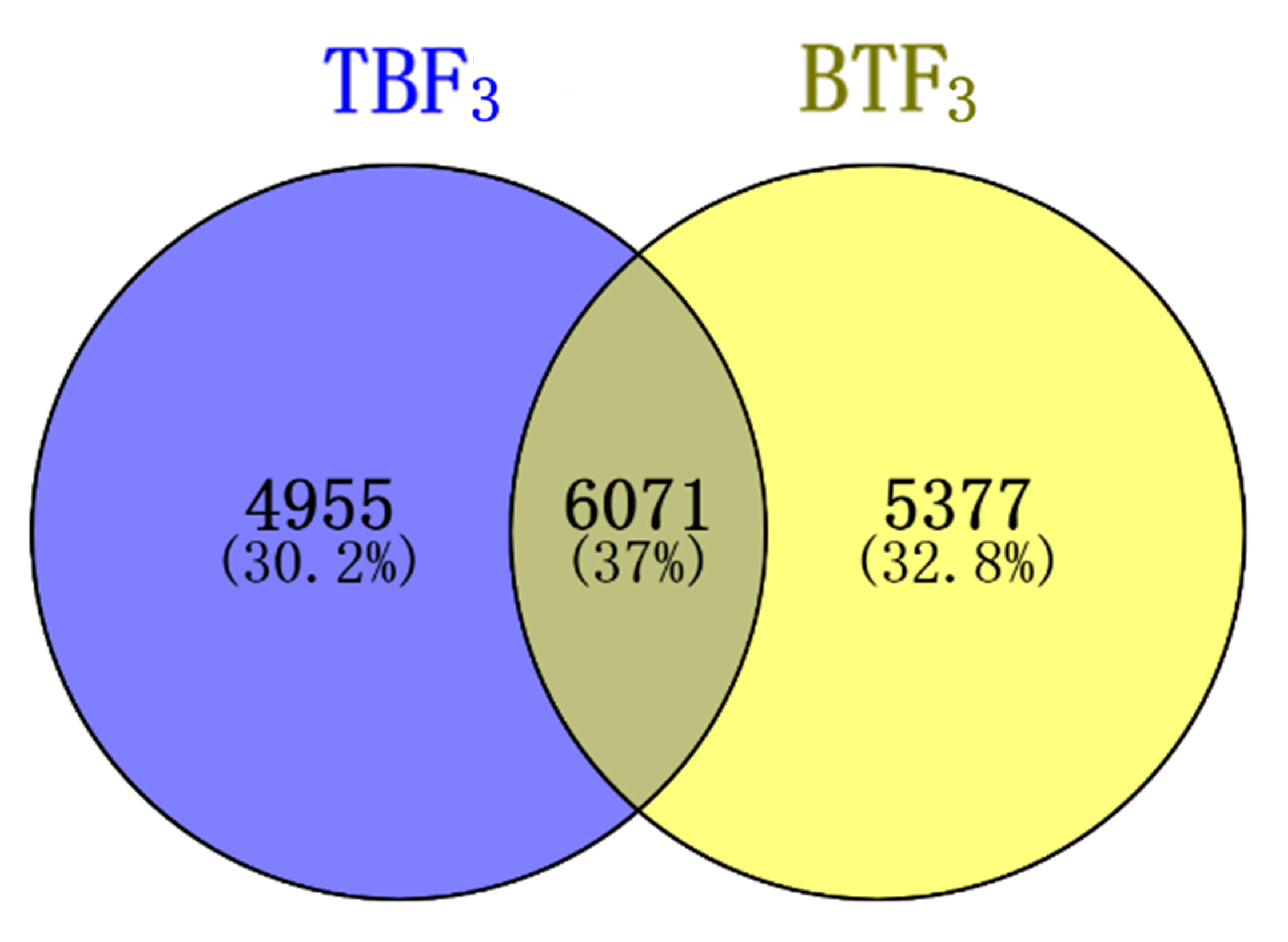

Supplement: Supplementary file 4 — Additional file 4: Figure S2. Shared genes between the two reciprocal cross hybrids detected by PacBio sequencing. [file 12864_2020_6866_MOESM4_ESM.tif]

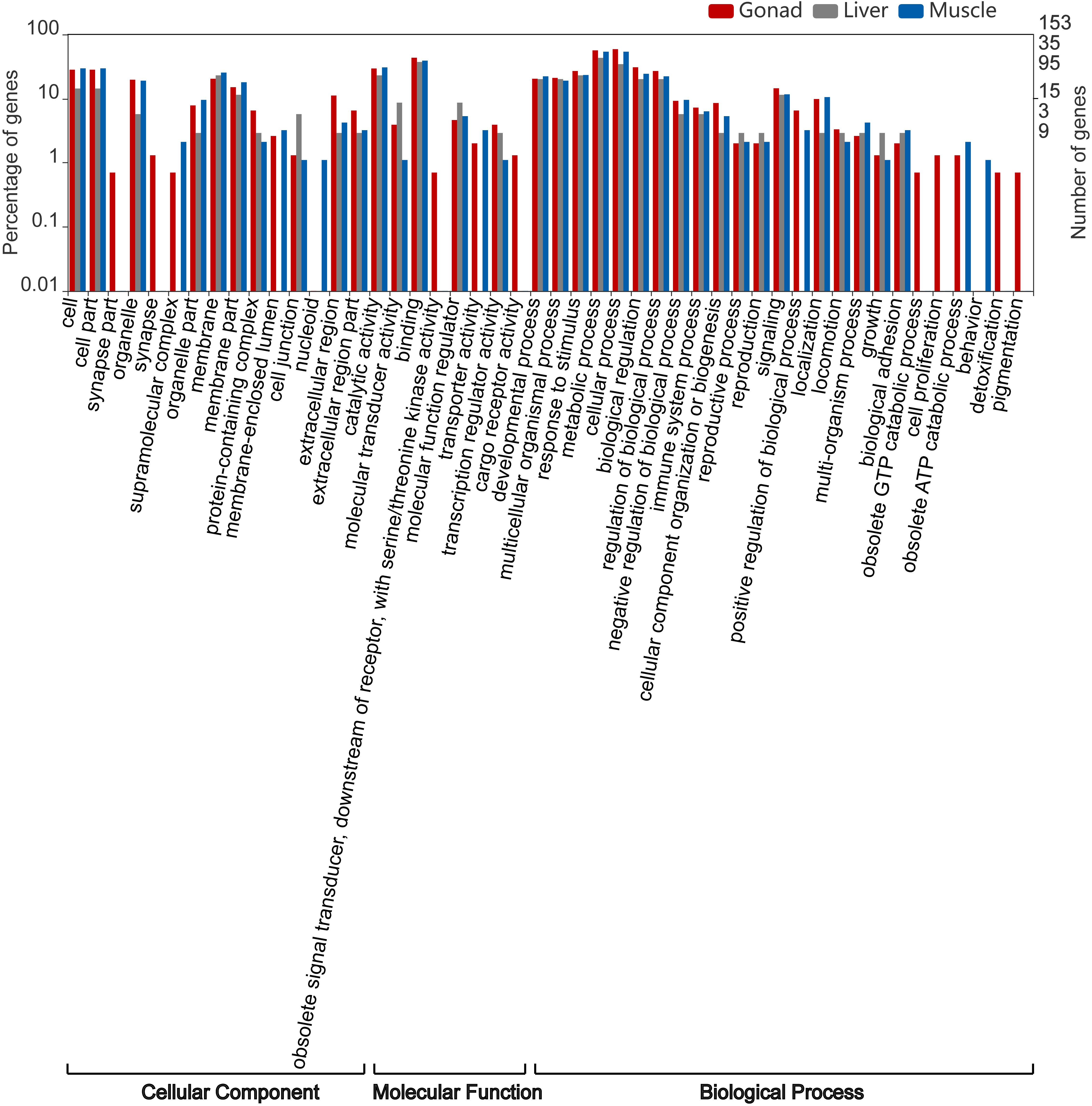

Supplement: Supplementary file 6 — Additional file 6: Figure S3. Gene ontology (GO) categories (level 2) of DEGs in gonad, liver, and muscle. [file 12864_2020_6866_MOESM6_ESM.tif]

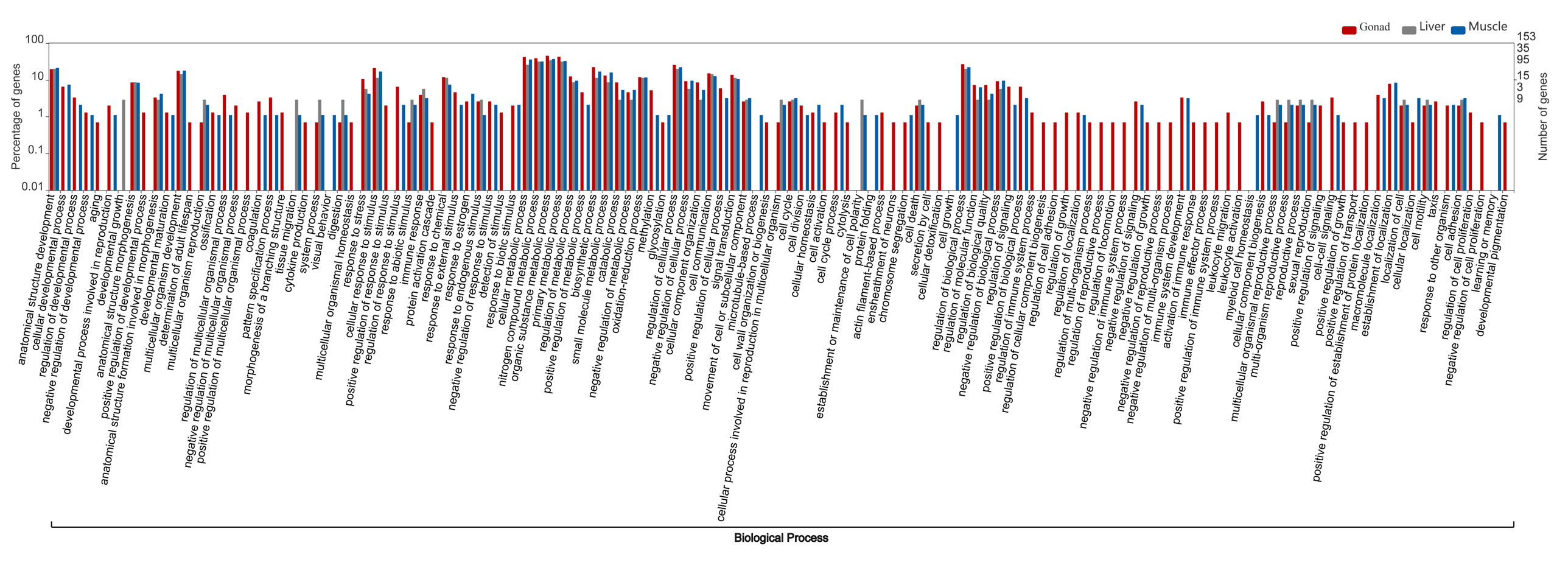

Supplement: Supplementary file 7 — Additional file 7: Figure S4. The distribution of DEGs in the biological process category (level 3) of gene ontology (GO). [file 12864_2020_6866_MOESM7_ESM.tif]

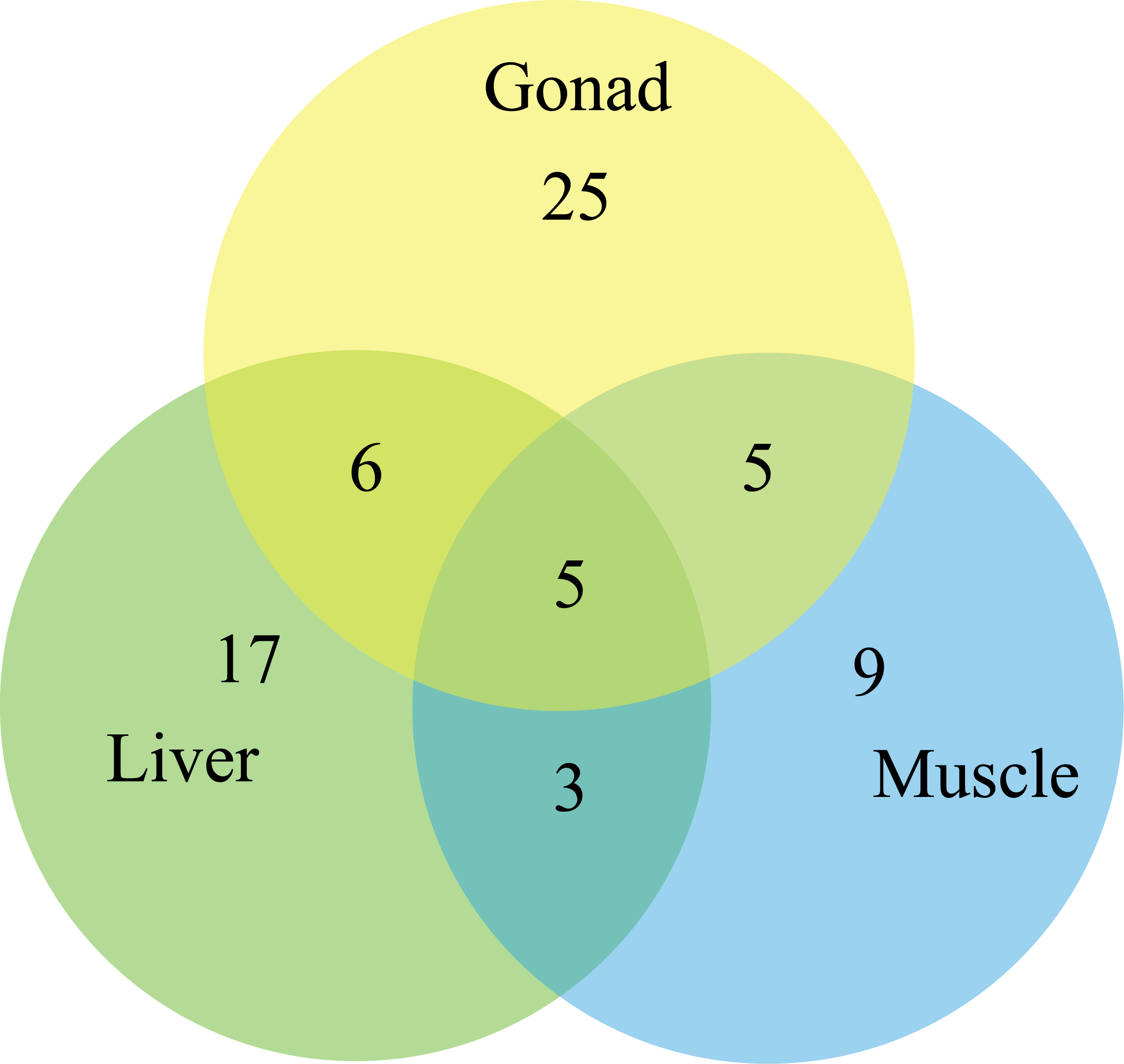

Supplement: Supplementary file 9 — Additional file 9: Figure S5. Distribution of genes with the high AS events (AS number ≥ 5) in gonad, liver, and muscle. [file 12864_2020_6866_MOESM9_ESM.tif]

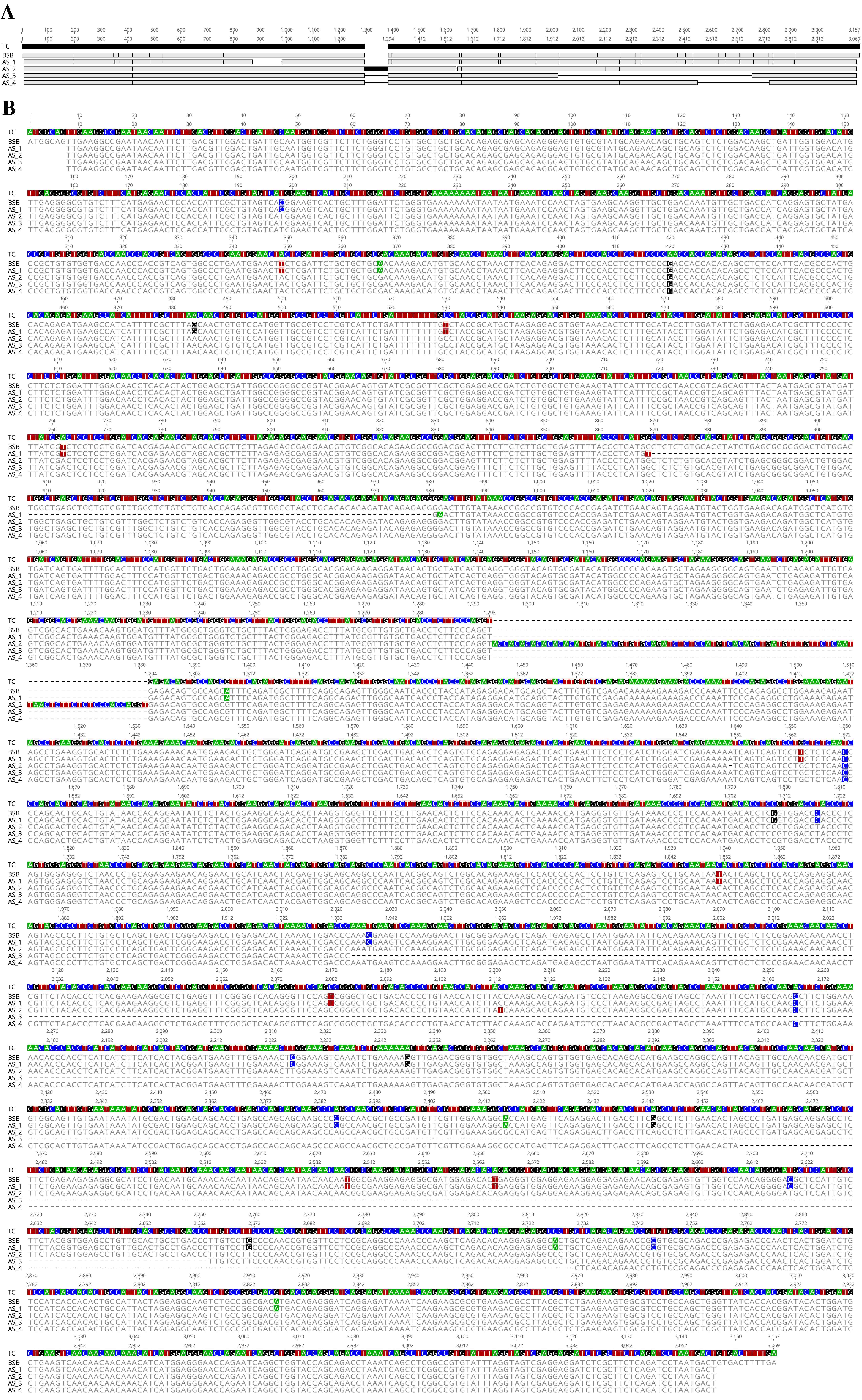

Supplement: Supplementary file 10 — Additional file 10: Figure S6. AS events of bmpr2a detected by Sanger sequencing. (A) The model of AS events in TBF3 and BTF3. TC and BSB represent the complete coding sequences in parental TC and BSB, respectively. AS_1 is the SE event in BSB-homoeologs, while AS_2, 3 and 4 are the AS events in TC-homoeologs. (B) The sequence alignments of the complete coding sequences in TC and BSB and the four AS in TBF3 and BTF3. [file 12864_2020_6866_MOESM10_ESM.jpg]
