# Supplementary material for: Maternal effects shape the alternative splicing of parental alleles in reciprocal cross hybrids of Megalobrama amblycephala × Culter alburnus
Source: BMC Genomics. 2020 Jul 2;21:457. doi: 10.1186/s12864-020-06866-7 (PMC7330940; doi:10.1186/s12864-020-06866-7)
Supplement: Supplementary file 8 — Additional file 8: Table S4. The AS events and their gene distribution predicted from Illumina data. [file 12864_2020_6866_MOESM8_ESM.docx]

**Additional file 7: Table S4**. The AS events and their gene distribution with predicted from Illumina data.

|  | Muscle | Liver | Gonad |
| --- | --- | --- | --- |
| MXE | 74 | 96 | 104 |
| SE | 2328 | 2863 | 3314 |
| Total AS events | 2402 | 2959 | 3418 |
| AS in TC-homoeologs | 2205 (91.80%) | 2706 (91.45%) | 3103 (90.78%) |
| AS in BSB-homoeologs | 197 (8.20%) | 253 (8.55%) | 315 (9.22%) |
| Gene NO. of AS in TC-homoeologs | 1707 (91.68%) | 2030 (91.03%) | 2371 (90.39%) |
| Gene NO. of AS in BSB-homoeologs | 155 (8.32%) | 200 (8.97%) | 252 (9.61%) |
| Total gene NO. of AS events | 1862 | 2230 | 2623 |

AS: Alternative splicing; SE: skipped exons; MXE: mutually exclusive exons.
